# Primary Care Physicians' Attitudes and Practices in Managing Overweight and Obesity in Al-Ahsa, Saudi Arabia

**DOI:** 10.7759/cureus.82670

**Published:** 2025-04-21

**Authors:** Mohammed A Alkhuwaysah, Hussain A Alsayegh, Maitham N Alsarhan

**Affiliations:** 1 Family Medicine, Alahsa Family Medicine Academy, Hofuf, SAU

**Keywords:** alahsa, attitude, attitude and practice of health care professionals, obesity, overweight, phc

## Abstract

Introduction: Obesity is a prevalent health issue that affects countries globally, regardless of their level of development. In Saudi Arabia, both male and female individuals are experiencing a growing concern with obesity, which begins in childhood and persists into adulthood. Primary healthcare doctors play a crucial role in addressing this problem, as they are the initial point of contact and significant contributors to cost-efficient strategies for managing and preventing obesity.

Aim: This study aims to determine the attitudes and practices of physicians working in primary health care centers regarding the management of overweight and obesity in Al-Ahsa, Saudi Arabia.

Methods: This was a descriptive cross-sectional study conducted in the Al-Ahsa region of Saudi Arabia. The study involved 188 participants by multistage stratified cluster sampling through two stages. The first stage involved randomly selecting 27 centers from 66 centers, proportional to the number of doctors in each sector. The second phase involved randomly selecting doctors at each selected center. Data were collected using a validated, pretested, self-administered questionnaire and analyzed by IBM SPSS Statistics for Windows, Version 28.0 (Released 2021; IBM Corp., Armonk, New York, United States). We employed both descriptive and inferential statistics.

Result: The study involved 188 participants, more than half (n=102, 54.3%) of whom were female, and 95 (50.5%) were aged 25-29 years. More than half (n=107, 56.9%) held board certificates, and 74 (39.4%) had bachelor's degrees. Over half (n=103, 54.8%) had less than three years of experience. A majority of participants (n=133, 70.70%) had a moderate attitude towards obesity, while 41 (21.8%) had a good attitude, and 14 (7.50%) had a bad attitude. Furthermore, more than two-thirds of them (67.50%) had moderate practice, while 37 (19.70%) had bad practice, and 24 (12.8%) had good practice. There was a significant relationship between sex and attitude, where male physicians had an attitude score higher than that of female physicians (29.86±3.43 vs 28.63±3.47) with a p-value of .016, and the study revealed a positive correlation between attitude and practice.

Conclusion: Improving the attitude and practices of primary care physicians towards the management of overweight and obesity will lead to better health outcomes in overweight and obese patients.

## Introduction

The World Health Organization (WHO) defined overweight as “a body mass index (BMI) of 25-29.9 kg/m^2^ and obesity as a BMI of ≥30 kg/m²” [1]. As a result of adopting Westernized lifestyles and behaviors with an excessive positive energy imbalance that has been compounded by a growing sedentary lifestyle in recent decades, obesity is the most prevalent nutritional disorder in industrialized countries, with an increase in childhood obesity [2,3].

WHO reported 2.5 billion adults in 2022 were overweight (43% of men and 44% of women), with over 890 million living with obesity [4]. According to data gathered from 79 of the 147 developing nations, 17.5 million children in these countries are estimated to be overweight. The Middle East, Latin America, and the Caribbean were determined to have the most significant rates of overweight people [5]. In Bahrain, females aged 2 to <4 years have higher overweight (12.3%) and obesity (8.4%), while males aged 4 to <6 years have higher overweight (8.4%) and obesity (7.2%) [6].

The National Health Survey in Saudi Arabia in 2023 indicated that approximately 24% of adults in the country were classified as obese, while the prevalence among children was 7.3%. However, there is no statistically significant difference between males and females, although the percentage of optimal weight was significantly higher in women (about 39.6%) than in men (about 29.5%) [7].

The management of obesity should mainly involve primary health care services. Guidelines for the management of obesity have been created in numerous countries since primary care physicians are essential to the proper assessment and treatment of obesity [8]. According to the United States Preventive Services Task Force, clinicians should refer or deliver intensive, multicomponent behavioral therapies to people with obesity [9].

Family physicians must determine whether a patient is ready to reduce weight and provide the necessary encouragement. A combination of a low-calorie diet, more exercise, and behavioral counseling is recommended for weight loss and maintenance [10]. To manage obesity in Saudi Arabia, it is essential to encourage physical exercise [11]. Primary health care physicians are the ideal source for health information [12,13]. Although it has been reported that dietitians can provide more affordable nutritional counseling for patients with obesity, the physicians could play a major role in the management of obesity [14, 15]. However, the counseling of patients with obesity is typically limited by a variety of issues [16,17], including a lack of dietitians, unmotivated and noncompliant patients, inadequate instructional materials, limited reimbursement, a low physician confidence level, and short consultation times [17,18]. Physicians have objectively observed or reported a lack of understanding of nutrition [19,20]. According to some theories, the primary causes of PHC's inadequate obesity management are doctors' ineffective intervention attempts and the low identification of patients' weight status [20]. A minority of individuals with obesity receive recommendations from their healthcare provider to engage in weight loss efforts [20]. The potential for better practice can be realized by supporting physicians with the necessary training and effectively addressing the constraints they face in their working environments [21].

Enhancing physician skill levels, especially in evaluating the extent of overweight, can be achieved through additional training in behavioral treatment approaches. Furthermore, training physicians in behavioral modification techniques may foster greater physician engagement in overweight prevention and treatment. The present study was conducted to determine the attitudes and practices of primary health care physicians in managing obesity and overweight in Al-Ahsa City, Eastern Saudi Arabia.

## Materials and methods

Study design and setting

This was a quantitative, descriptive, cross-sectional, questionnaire-based study conducted from August 2024 to January 2025. The study was conducted in the Al-Ahsa region, which accounts for about 20% of the area of Saudi Arabia. It is considered the largest governorate in the Eastern Province of Saudi Arabia. It contains four major cities: Al Hofuf, Al Mubarraz, Al Oyun, and Al Umran. The total population of Al-Ahsa is about 1,369,338. Al-Ahsa is divided into four health clusters: Southern cluster, Middle cluster, Northern cluster, and Eastern cluster. The total number of primary health care centers in all the clusters is 66 [22].

Study population

All primary health care physicians working at Ministry of Health (MOH) primary health care centers in Al-Ahsa, including general practitioners, trainees, specialists, and consultants, were included in the study. Physicians who worked in urgent care centers and antenatal care were excluded.

Sampling technique

The sample size was calculated using the RaoSoft software (Raosoft Inc., Seattle, Washington, United States) with a confidence level of 95% and a margin of error set at 5%. The initial calculated sample size was determined to be 166 participants, considering a total population of 501 individuals and a prevalence rate based on a prior study (20%). Recognizing the importance of study power and potential missing data, we increased our sample size by an additional 12%. Therefore, the final targeted sample size for our investigation was 188 participants.

Total Population Size

We employed a multistage cluster sampling method to ensure the reliability and relevance of our study results, minimize errors and biases, and enhance statistical power. The study included all primary care doctors in the Al-Ahsa area (N=501), who worked at 66 primary health care centers (PHCCs) spread across four different regions: Eastern (19 centers, 110 doctors), Northern (16 centers, 107 doctors), Southern (20 centers, 88 doctors), and Middle (11 centers, 99 doctors). The population also included 92 primary physician trainees at the Al-Ahsa Family Medicine Academy and five physicians serving in administrative roles across these sectors. The sampling process involved two stages. In the first stage (cluster sampling), 27 PHCCs were randomly selected from the total of 66 centers. The second stage involved the application of simple random sampling to recruit participants from within each of the selected centers.

Data collection

Data were collected by a validated, pretested, self-administered questionnaire designed to assess the attitudes and practices of physician assistants regarding obesity management in a previous study after due permission [23]. The questionnaire consisted of three parts: the first part was personal data (age, gender, marital status, nationality, language, management, and exercise counselling); the second part involved 13 statements measuring physician attitudes, which were scored by a three-point Likert scale (3-agree, 2-neutral, and 1-disagree); and the third part contained 17 statements measuring physicians' practices in obesity management, which were scored by a three-point Likert scale (3-always, 2-sometimes, and 1-never). The internal consistency of the items was tested using a Cronbach alpha reliability test at .85, and content validity was confirmed by experts.

Statistical analysis

IBM SPSS Statistics for Windows, Version 28.0 (Released 2021; IBM Corp., Armonk, New York, United States) was used for statistical analysis. Descriptive statistics (means, standard deviations, frequencies, and percentages) were calculated. An independent t-test and ANOVA test were used to test the relationship between sociodemographic factors and attitude and practice. Pearson correlation was used to find the correlation between attitude and practice. A P value ≤ 0.05 was considered statistically significant in all tests.

## Results

The study included 188 participants, of whom more than half were female (n=102, 54.3%) and aged 25-29 years (n=95, 50.5%). About three-quarters (n=140, 74.5%) were married. Regarding educational qualifications, more than half (n=107, 56.9%) held board certificates, while 74 (39.4%) had bachelor's degrees. Furthermore, more than half of them (n=103, 54.8%) had less than three years of experience. More details regarding sociodemographic characteristics are in Table 1. 

**Table 1:** Sociodemographic characteristics of the participants (N=188)

Characteristics		Frequency	Percentage
Age groups (years)	25-29	95	50.5
	30-34	54	28.7
	35-39	29	15.4
	40-44	5	2.7
	≥45	5	2.7
Sex	Male	86	45.7
	Female	102	54.3
Marital status	Married	140	74.5
	Single	44	23.4
	Divorced	4	2.1
Highest qualification degrees	Bachelor	74	39.4
	Diploma	1	.5
	Master	3	1.6
	Board	107	56.9
	Fellowship	3	1.6
Years of experience	< 3	103	54.8
	3 to < 6	40	21.3
	6 to < 9	16	8.5
	≥ 9	29	15.4

Table 2 shows that the majority of participants (n=181, 96.3%) agreed that obesity is considered a disease. Similarly, 181 (96.3%) also believed that even small weight loss can produce health benefits for overweight and obese individuals. Furthermore, 158 (84.0%) participants agreed that treating overweight and obese people is professionally rewarding. Additionally, 143 (76.1%) agreed that physicians should serve as models in maintaining a normal weight. In terms of stereotypes, 113 (60.1%) participants agreed that overweight people tend to be lazier than those of normal weight. Furthermore, 100 (53.2%) believed that overweight individuals lacked willpower and motivation in comparison with people of normal weight. More than one-quarter of the participants (n=48, 25.5%) believed that primary health care centers were well-prepared to manage overweight and obesity. On the other hand, only 38 (20.2%) agreed that physicians should refer overweight and obese patients to other professionals rather than treat them themselves. Regarding self-identification, 138 (73.4%) participants didn’t consider themselves obese, and 104 (55.3%) didn’t consider themselves overweight.

**Table 2:** Attitude of participants with regard to obesity (N=188)

No	Items		Agree	Neutral	Disagree	Mean	SD
1	Obesity is considered as a disease	Frequency	181	4	3	2.95	.29
		Percentage	96.3	2.1	1.6		
2	Overweight people tend to be lazier than people with normal weight	Frequency	113	54	21	2.49	.69
		Percentage	60.1	28.7	11.2		
3	Overweight people lack willpower and motivation in comparison with normal-weight people	Frequency	100	58	30	2.37	.75
		Percentage	53.2	30.9	16.0		
4	Physicians' role is to refer overweight and obese patients to other professionals rather than attempt to treat them	Frequency	38	40	110	1.62	.80
		Percentage	20.2	21.3	58.5		
5	Counseling in weight reduction is easy	Frequency	38	61	89	1.73	.78
		Percentage	20.2	32.4	47.3		
6	For overweight and obese people, even small weight loss can produce health benefits	Frequency	181	7	0	2.96	.19
		Percentage	96.3	3.7	0		
7	Physician should be models in maintaining normal weight	Frequency	143	35	10	2.71	.56
		Percentage	76.1	18.6	5.3		
8	Treating overweight and obese people is professionally rewarding	Frequency	158	27	3	2.82	.42
		Percentage	84.0	14.4	1.6		
9	Only a small percentage of overweight and obese people can lose weight and maintain this loss	Frequency	69	69	50	2.10	.79
		Percentage	36.7	36.7	26.6		
10	I feel confident in managing overweight and obese patients	Frequency	90	74	24	2.35	.70
		Percentage	47.9	39.4	12.8		
11	I consider myself obese	Frequency	28	22	138	1.41	.74
		Percentage	14.9	11.7	73.4		
12	I consider myself overweight	Frequency	67	17	104	1.80	.94
		Percentage	35.6	9.0	55.3		
13	I feel that primary health care centers are well prepared to manage overweight and obesity	Frequency	48	68	72	1.87	.79
		Percentage	25.5	36.2	38.3		

Table 3 shows that the majority of participants (n=166, 88.3%) always advise their patients to increase physical activities to reduce weight. Additionally, 158 (84.0%) always provide weight control advice for patients with chronic illnesses such as diabetes or dyslipidemia. Also, 134 ( 71.3%) participants always advise patients to reduce caloric intake for weight loss. In diagnostic practices, 180 (95.7%) participants always use BMI to assess overweight or obesity. Conversely, only 25 (13.3%) always use waist circumference, 23 (12.2 %) use appearance to diagnose overweight or obesity, and 13 (9.0%) use waist-hip ratio. Furthermore, 133 (70.7%) always refer their obese patients to dietitians for specialized nutritional support, 92 (48.9%) participants always refer obese patients for surgery when indicated, and 45 (23.9%) refer them for behavioral therapy. Regarding treatment, only 14 (7.4%) always prescribe weight-reducing medications, and 27 (14.4%) always record food intake diaries for obese patients. More than one-third of participants (n=72, 38.3%) provide educational materials as part of managing overweight or obesity, and just 19 (10.1%) have a group support system for obese patients.

**Table 3:** Practice followed by participants with regard to obesity (N=188)

No	Items		Always	Sometimes	Never	mean	SD
1	Do you advice your patients to increase physical activities to reduce their weight?	Frequency	166	22	0	2.88	.32
		Percentage	88.3	11.7	0		
2	Do you advise your patients to reduce caloric intake to reduce their weight?	Frequency	134	50	4	2.69	.51
		Percentage	71.3	26.6	2.1		
3	Do you use "weight without height" to diagnose overweight or obesity?	Frequency	13	18	157	1.23	.57
		Percentage	6.9	9.6	83.5		
4	Do you use "Body Mass Index (BMI)" to diagnose overweight or obesity?	Frequency	180	8	0	2.96	.20
		Percentage	95.7	4.3	0		
5	Do you use "Waist circumference" to diagnose overweight or obesity?	Frequency	25	76	87	1.67	.70
		Percentage	13.3	40.4	46.3		
6	Do you use "Waist-Hip ratio" to diagnose overweight or obesity?	Frequency	17	48	123	1.44	.66
		Percentage	9.0	25.5	65.4		
7	Do you use "Appearance" to diagnose overweight or obesity?	Frequency	23	81	84	1.68	.68
		Percentage	12.2	43.1	44.7		
8	Do you refer your obese patients to dietitians in obesity management?	Frequency	133	55	0	2.71	.46
		Percentage	70.7	29.3	0		
9	Do you refer your obese patients for behavioral therapy in obesity management?	Frequency	45	58	85	1.79	.81
		Percentage	23.9	30.9	45.2		
10	Do you refer your obese patients for surgery if indicated?	Frequency	92	92	4	2.47	.54
		Percentage	48.9	48.9	2.1		
11	Do you prescribe weight-reducing medications?	Frequency	14	58	116	1.46	.63
		Percentage	7.4	30.9	61.7		
12	Do you provide educational materials as part of managing overweight or obesity?	Frequency	72	69	47	2.13	.79
		Percentage	38.3	36.7	25.0		
13	In your practice, do you have "a group support" for obese patients?	Frequency	19	28	141	1.35	.66
		Percentage	10.1	14.9	75.0		
14	Do you offer weight control advice for your patients with chronic illness, e.g., diabetes or dyslipidemia, as part of the management?	Frequency	158	26	4	2.82	.44
		Percentage	84.0	13.8	2.1		
15	Do you advice food intake diary for obese patients?	Frequency	27	64	97	1.63	.72
		Percentage	14.4	34.0	51.6		
16	Do you refer obese patients to physical exercise practitioners?	Frequency	52	55	81	1.85	.83
		Percentage	27.7	29.3	43.1		

Table 4 reveals that there was no significant relationship between age, marital status, qualification degree, and years of experience with attitude. However, there was a significant relationship between sex and attitude, where male participants had an attitude score (29.86±3.43) higher than female participants (28.63±3.47) with a p-value of .016.

**Table 4:** Relationship between sociodemographic characteristics and attitude ^a ^AONVA test; ^b^ independent t test; ^*^significant

Sociodemographic characteristics		Frequency	Mean attitude score	SD	P value
Age group (years) ^a^	25-29	95	28.92	4.10	.675
	30-34	54	29.76	2.72	
	35-39	29	29.10	2.88	
	40-44	5	29.60	3.13	
	≥45	5	28.40	1.82	
Sex ^b^	Male	86	29.86	3.43	.016*
	Female	102	28.63	3.47	
Marital status ^a^	Married	140	29.23	3.29	.535
	Single	44	29.25	4.17	
	Divorced	4	27.25	2.75	
Highest qualification degrees ^a^	Bachelor	74	29.09	4.14	.979
	Diploma	1	29.00	.	
	Master	3	30.00	3.00	
	Board	107	29.26	3.08	
	Fellowship	3	28.33	2.08	
Years of experience ^a^	less than 3 years	103	29.28	3.96	.799
	3 – less than 6 years	40	28.88	3.10	
	6 – less than 9 years	16	29.81	2.74	
	9 years or more	29	28.97	2.60	

Table 5 shows there was no significant relationship between age, sex, marital status, qualification degree, and years of experience with practice.

**Table 5:** Relationship between sociodemographic characteristics and practice a: AONVA test, b: independent t test

Sociodemographic characteristics		N	Mean practice score	SD	P value
Age ^a^	25-29	95	32.54	5.53	.460
	30-34	54	32.41	4.69	
	35-39	29	34.00	4.43	
	40-44	5	31.00	2.55	
	≥45	5	34.80	2.68	
Sex ^b^	Male	86	33.47	4.67	.071
	Female	102	32.14	5.26	
Marital status ^a^	Married	140	32.65	4.99	.314
	Single	44	33.34	5.27	
	Divorced	4	29.50	3.00	
Highest qualifications degrees ^a^	Bachelor	74	33.01	5.89	.749
	Diploma	1	34.00	.	
	Master	3	34.67	5.51	
	Board	107	32.42	4.43	
	Fellowship	3	35.33	3.06	
Years of experience ^a^	less than 3 years	103	32.61	5.63	.647
	3 – less than 6 years	40	32.67	4.27	
	6 – less than 9 years	16	31.94	4.96	
	9 years or more	29	33.76	3.70	

**Figure 1:**
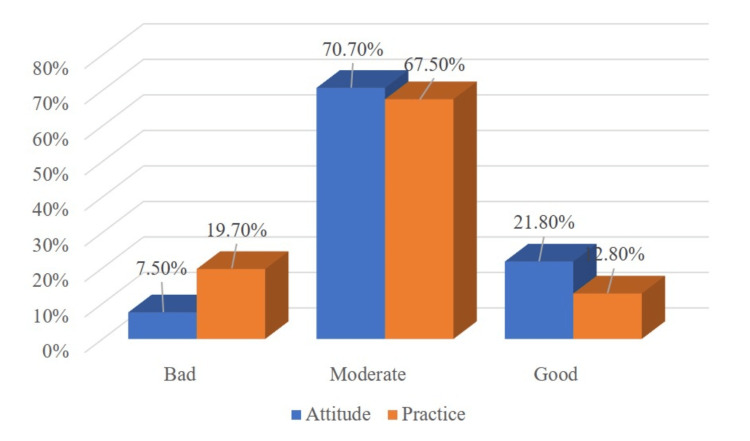
Attitude and practice scores with regard to obesity

Figure 1 shows that the majority of participants (n=133, 70.70%) had a moderate attitude towards obesity, while 41 (21.8%) had a good attitude, and 14 (7.50%) had a bad attitude. Furthermore, more than two-thirds (67.50%) had moderate practice, while 37 (19.70%) had bad practice, and 24 (12.8%) had good practice.

Table 6 shows that there was a positive correlation between attitude and practice with Pearson’s r (.480) and P. value <.001.

**Table 6:** Correlation between attitude and practice

Attitude & practice	Pearson’s r	P. Value
	.480	< 0.001

## Discussion

This was a descriptive cross-sectional study aimed to determine the attitudes and practices of primary health care physicians in managing obesity in Alahsa City, Eastern Saudi Arabia.

The current study revealed that 70% of our physicians had a moderate attitude toward obesity, 21% had a good attitude and only 7% had a bad attitude revealing that our physicians had a positive attitude toward obesity. This finding was in the same line with previous studies in Saudi Arabia and Namibia, which revealed 44% and 70% of their physicians had good and positive attitudes, respectively [24,25]. The vast majority of physicians in the current study (96%) agreed that obesity is considered a disease. This consideration is slightly higher than in two previous studies conducted in Saudi Arabia, where 90% and 86% of their physicians considered obesity as a disease [23,26]. Studies in Bahrain and Iraq reported that most of their physicians considered obesity as a great health problem in their country [27,28]. On the other hand, two recent studies in Tabuk (KSA) and India reported that around 71% and 57% considered obesity as a chronic disease [29,30].The study notes that while physicians recognize obesity as a disease, their actual practice does not always align with this belief. These variations could be due to barriers such as time constraints, lack of patient motivation, or healthcare system limitations.

In the current research, 60% of physicians believed that individuals with overweight conditions are more likely to exhibit laziness compared to those of normal weight, and they perceived them as lacking willpower and motivation. Therefore, 20% of the respondents indicated that providing counseling to these patients would be easy. These findings were supported by studies in Saudi Arabia and Hungary [23,31].

Only 20% of our participants agreed that physicians should refer overweight and obese patients to other professionals rather than treat them, which had the lowest attitude mean score. Furthermore, 70% and 49% of physicians used to refer their obese patients to dietitians and surgery to manage obesity. Manjunath et al. reported that around 41% of their general practitioners disagreed with referring overweight and obese persons to specialists instead of attempting to treat them, while 60% of them felt that they were professionally well trained to treat overweight or obese people [30]. A recent Saudi study reported that around 36% of their physicians reported having no prior experience in managing obesity, while 21% indicated that they encountered challenges in facilitating a referral [29]. On the other hand, a study in Italy revealed that around 74% of their physicians had referred their patients to bariatric surgeons [32]. A Swedish study revealed that their physicians experienced a sense of frustration in their efforts to assist obese individuals and referred them to undergo bariatric surgery [33].

Lifestyle management was the most prominent method used by our physicians, in which the majority of our participants indicated that they consistently recommend to their patients the enhancement of physical activity and the reduction of caloric intake in order to achieve weight loss. Consistently, several studies in Saudi Arabia, Bahrain, and Hungary reported that lifestyle modification was the highest-practiced method among their physicians [23,26,27,29,31]. Many primary care physicians are open to discussing lifestyle modification strategies with their patients. The Saudi Ministry of Health's clinical practice guidelines recommend comprehensive lifestyle changes, including dietary adjustments, increased physical activity, and behavioral therapy. A study of Australian general practitioners found that most GPs considered training in dietary and physical activity advice for overweight patients important but felt limited by their insufficient nutrition knowledge and obesity counseling skills [34].

The majority of our participants indicated that they rely on BMI for diagnosing obesity; however, there was a noticeable gap in the use of alternative methods. Specifically, 13% of participants consistently employed waist circumference measurements, while approximately 67% reported never using the waist-to-hip ratio. These results were better than those of a study in Dammam and Al-Khobar, where 60%, 12%, and 10% of the people who took part used BMI, waist circumference, and waist-hip ratio, respectively, to diagnose obesity [23]. Additionally, a recent survey conducted in Sweden among 1642 primary care physicians reported that, although almost all of their participants use BMI measurements, only 35% and 2% use waist circumference and waist-hip ratio [35]. A study in Hungary found that BMI measurement was mostly used among physicians in cities rather than rural areas and waist circumference and waist-hip ratio were mostly used for pediatrics rather than adults [31].

Around 61% of our physicians have never prescribed medications for weight reduction, while 38% incorporate educational resources into their approach to managing obesity. A Saudi study revealed that overweight or obese physicians tended to exhibit greater comfort in prescribing weight loss medications to obese patients [36]. Moreover, an Iraqi study reported that three-quarters of their physicians prescribe drugs to obese patients when their BMI > 30 kg/m² [28]. Inconsistently, studies in Kuwait and Korea revealed only lower percentages than this study [37,38].

In our study, there was a significant correlation between the attitude score and the gender of physicians, in which male physicians showed a higher mean attitude than female physicians. This finding was irrelevant to a study conducted in Germany where females were having a higher positive attitude score than males [39]. Foster et al. mentioned that female physicians had higher attitude and practice scores regarding obesity [12]. Alatawi et al. mentioned that the level of comprehensive diet counseling was notably higher among female physicians [29]. Possible explanations for the variance in attitude scores between male and female physicians are likely influenced by a combination of socialization, different study tools, professional experiences, patient interactions, and broader cultural factors.

Strengths and limitations of the study

Employing multistage, stratified cluster sampling improves the sample's representativeness and provides a more precise representation of the attitudes and practices of primary healthcare providers across several centres in Al-Ahsa. Also, the data collection employed validated and pretested self-administered questionnaires, enhancing the reliability and validity of the results. Moreover, the study's sample size of 188 participants provides considerable statistical power. However, there were some limitations, such as the study's cross-sectional design. The use of self-reported data may introduce bias. There may be differences in culture and systems that make the results not applicable to other parts of Saudi Arabia or to other healthcare settings, since the study only looked at Al-Ahsa.

## Conclusions

The study reveals that a majority of participants had a moderate attitude towards obesity, and more than two-thirds of them had moderate practice. Also, male participants had a higher attitude score than female participants. Subsequently, specialized training programs to address the identified gaps are necessary for primary healthcare providers to improve their attitudes and practices regarding obesity management through workshops, seminars, and continuing medical education based on evidence-based medicine. Furthermore, future studies should investigate the factors influencing attitudes and practices, as well as the long-term impacts of training interventions on physician behavior. Further research on physician barriers and patient engagement strategies is recommended as well.
